# Effects of Different Cooking Methods (Pan‐Frying, Sous‐Vide, Boiling, and Deep Frying) on Mineral Composition and In Vitro Bioaccessibility of Beef, Lamb, and Chicken Livers

**DOI:** 10.1002/fsn3.72011

**Published:** 2026-06-10

**Authors:** Ayşe Semra Aksoy, Hatice Bekiroglu, Muhammet Arici, Osman Sagdic

**Affiliations:** ^1^ Department of Nutrition and Dietetics, Faculty of Health Sciences Bezmialem Vakif University Istanbul Turkiye; ^2^ Department of Food Engineering, Faculty of Agriculture Sirnak University Sirnak Turkiye; ^3^ Department of Food Engineering, Faculty of Chemical and Metallurgical Engineering Yildiz Technical University Istanbul Turkiye

**Keywords:** bioaccessibility, edible liver, ICP‐OES, in vitro digestion, minerals, soluble fraction, thermal processing

## Abstract

This study investigated the effects of pan‐frying, sous‐vide, boiling, and deep frying methods on the mineral content and post–in vitro digestion bioaccessibility of beef, lamb, and chicken livers. Sodium (Na), magnesium (Mg), potassium (K), phosphorus (P), iron (Fe), copper (Cu), manganese (Mn), zinc (Zn), chromium (Cr), and cobalt (Co) concentrations were quantified using inductively coupled plasma atomic emission spectrometry (ICP‐OES). Significant differences in mineral contents were observed among cooking methods within the same liver type and among liver types within the same cooking method (*p* < 0.05). Sous‐vide achieved the highest macromineral retention across all liver types, ranging from 61.52% ± 1.83% to 101.45% ± 6.21%. Micromineral retention varied by liver type, with boiling and sous‐vide methods yielding higher rates (39.69% ± 2.84%–141.05% ± 9.93%). Sous‐vide treatment was also associated with an apparent post‐processing increase in Mg concentration (29.82%), reflecting high mineral retention efficiency and reduced moisture loss rather than de novo mineral formation. This cooking method also resulted in the highest Fe concentrations (281.6 ± 7.86 mg/kg). In contrast, boiling caused losses of 49.24%–70.69% in Na, Mg, K, and P, while deep frying resulted in losses of 3.38%–48.90% for these elements. After in vitro digestion, boiling resulted in the lowest mineral losses across all liver types. The samples exhibiting the most pronounced post‐digestion increases and the highest Fe concentrations were identified as 560.44 ± 35.52 mg/kg for BFI (Beef Deep Frying Intestine), 636.4 ± 12.93 mg/kg for CFI (Chicken Deep Frying Intestine), and 728.4 ± 27.09 mg/kg for LBI (Lamb Boiling Intestine). Overall, considering both mineral retention after cooking and mineral release into the soluble fraction during in vitro digestion, sous‐vide emerged as the most favorable cooking method for preserving the mineral nutritional quality of liver, while the findings should still be interpreted in the context of bioaccessibility and realistic dietary intake.

## Graphical abstract

Beef, lamb, and chicken livers were subjected to pan‐frying, sous‐vide, boiling, and deep frying, followed by simulated gastric and intestinal digestion. Mineral concentrations and soluble fractions were quantified by ICP‐OES to evaluate cooking‐related mineral retention and in vitro bioaccessibility. Overall, sous‐vide emerged as the most favorable method for preserving the mineral nutritional quality of liver.

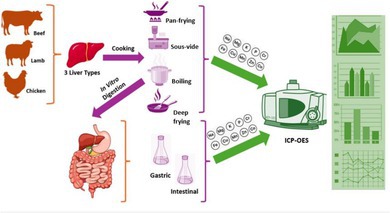

## 1 Introduction

Meat is an important component of the human diet because it provides high‐quality protein and several essential micronutrients, including minerals. Among meat‐derived foods, liver is considered a nutrient‐dense edible offal due to its notable contents of iron, zinc, copper, phosphorus, and other minerals. Therefore, evaluating the mineral composition of liver is important for understanding its potential contribution to dietary mineral intake. Minerals obtained through dietary intake are essential micronutrients, and adequate and balanced mineral consumption plays a critical role in maintaining normal human health and preventing diseases. Inadequate mineral intake may result from insufficient dietary consumption or from absorption disorders, which can be associated with infections or chronic inflammation (Kiani et al. 2022). Mineral deficiencies remain a significant public health challenge; in The State of Food Security and Nutrition in the World 2025 report, the Food and Agriculture Organization (FAO) identifies this issue as a key component of the global nutrition crisis, while the European Food Safety Authority (EFSA), in its 2024 Annual Report on Emerging Risks and Horizon Scanning Activities, classifies trace mineral deficiencies under the “emerging risk” category and emphasizes the need to monitor public health risks associated with biological hazards (EFSA 2025; FAO 2025). In general, minerals account for only 5% of a typical human diet (Goluch et al. 2025). Although a balanced diet generally provides adequate minerals, understanding their specific roles and ensuring sufficient intake are essential for maintaining overall health and preventing diseases (Razzaque and Wimalawansa 2025).

In the human diet, meat serves as a key provider not only of high‐quality proteins but also of essential minerals, and notably, organ meats such as liver may supply up to 50% of the Recommended Dietary Allowance (RDA) for specific minerals (Pereira et al. 2013; Tomović et al. 2011; Wood 2017). In this context, mineral composition data are often interpreted within a practical dietary framework rather than under strictly standardized compositional equivalence. Accordingly, the present approach was chosen to emphasize practical dietary relevance rather than purely compositional equivalence. Observed interspecies differences are therefore considered intrinsic biological characteristics rather than experimental bias, and they provide meaningful insight into species‐specific nutritional profiles of liver. Beyond its nutritional value, the evaluation and use of liver and other edible offal may also contribute to more sustainable meat production by improving carcass utilization and reducing the loss of nutrient‐dense animal‐derived foods (Latoch et al. 2024).

Because liver and many other fresh meat products are generally consumed after cooking, thermal processing is important for improving palatability, texture, and microbial safety (Domingo 2011; Tornberg 2005). Cooking can not only enhance sensory attributes such as texture, tenderness, and flavor, but also alter the chemical form of nutrients, thereby influencing their bioaccessibility. Since many minerals are associated with proteins in meat or are directly incorporated into the protein structure, the bioavailability of these nutrients to the human body may vary depending on the cooking technique employed (Da Silva et al. 2017). Thermal processing induces unfavorable alterations in meat functionality, including protein fraction denaturation, lipid oxidation, and the depletion of specific vitamins and minerals (Wereńska et al. 2023). Moreover, certain cooking methods applied at high temperatures may lead to the formation of compounds harmful to human health, such as heterocyclic aromatic amines and polycyclic aromatic hydrocarbons (Goluch et al. 2021; Modzelewska‐Kapituła et al. 2019; Suleman et al. 2019; Wereńska et al. 2021; Woloszyn et al. 2020). These chemical and structural changes depend on thermal processing parameters, including cooking environment, temperature, and duration, with each method distinctly affects nutrient bioavailability (Combes et al. 2004; Da Silva et al. 2017). Cooking procedures may modify the mineral profile of meat in two primary ways. A reduction in water content can increase the dry matter proportion of meat, thereby concentrating nutrients, including chemical elements (Czerwonka and Szterk 2015; Lombardi‐Boccia et al. 2005; Purchas et al. 2014). On the other hand, with water loss, meat also loses valuable water‐soluble compounds, which may lead to a reduction in its mineral element content (Gharibzahedi and Jafari 2017; Oz et al. 2017). Beyond total mineral concentration, the nutritional relevance of cooked liver depends on the fraction of minerals released into the soluble phase during gastrointestinal digestion. Cooking methods may influence this process through moisture loss, protein denaturation, tissue disruption, and the transfer of water‐soluble minerals into the cooking medium. Recent studies have emphasized that thermal processing can alter mineral bioaccessibility by changing food matrix structure and the release of minerals during digestion. Rebellato et al. (2022) evaluated the bioaccessibility of Fe, Zn, Ca, and Mg in processed pork and beef products using static in vitro digestion methods, highlighting the importance of assessing minerals beyond their total concentration. Similarly, García‐Conde et al. (2025) reported that Fe bioaccessibility depends on food type and preparation method, supporting the need to consider cooking conditions when evaluating mineral availability. In vitro digestion models are therefore useful tools for estimating the soluble mineral fraction potentially available for intestinal absorption, although they should be interpreted as indicators of bioaccessibility rather than direct measures of physiological bioavailability (Ośko et al. 2024). In this context, Da Silva et al. (2017) specifically showed that heat treatments can increase the bioaccessibility of several minerals in bovine liver, with sous‐vide generally providing higher bioaccessibility than boiling, except for potassium. Accordingly, considering the bidirectional effects of cooking on mineral retention and mineral release during digestion, both the dietary source of the mineral and the applied cooking method should be considered when evaluating the potential nutritional contribution of liver. Although previous studies have reported the mineral composition of meat and edible offal, limited data are available on how different cooking methods affect both mineral content and the release of minerals into the soluble intestinal fraction of beef, lamb, and chicken livers under comparable experimental conditions. Accordingly, this study aimed to investigate changes in sodium (Na), magnesium (Mg), potassium (K), phosphorus (P), iron (Fe), copper (Cu), manganese (Mn), zinc (Zn), chromium (Cr), and cobalt (Co) contents in beef, lamb, and chicken liver samples subjected to different cooking methods (pan‐frying, boiling, deep frying, and sous‐vide), and subsequently to assess the in vitro bioaccessibility of these minerals based on their soluble intestinal fraction. Thus, the study seeks to elucidate the relationships between changes in mineral content induced by cooking and the subsequent release of minerals into the digestive fluid during in vitro digestion, as influenced by cooking methods and liver type, thereby contributing to more informed dietary choices. Moreover, this study presents a novel contribution to the literature by comparatively evaluating three different species (beef, lamb, and chicken liver) under identical cooking conditions while simultaneously providing a comprehensive assessment of mineral content and in vitro bioaccessibility.

## 2 Materials and Methods

### 2.1 Liver Samples

Fresh beef, lamb, and chicken liver samples used in this study were obtained from local commercial butchers in Istanbul, Türkiye, who sourced their products from animals slaughtered in commercial abattoirs operating in compliance with the Turkish National Standard. Approximately 1 kg of liver from each animal species was purchased on the same day. Information regarding the sex of the animals was not available at the point of purchase. All samples were transported to the laboratory under cold‐chain conditions to preserve their freshness and stored at −20°C for a maximum of 24 h prior to cooking and subsequent analyses. Before sample preparation, the frozen samples were thawed under controlled conditions at +4°C in a refrigerator to maintain sample integrity and prevent quality deterioration.

### 2.2 Chemicals

Pepsin (from porcine gastric mucosa, P7000, activity > 250 U/mg), α‐amylase from the porcine pancreas (Type IV‐B, A3176), α‐glycosidase enzyme from *Saccharomyces cerevisiae* (Type I, G5003), and pancreatin (from porcine pancreas, P7545, 8 × USP specifications) were obtained from Sigma‐Aldrich (St. Louis, MO, USA). Invertase (from yeast, 300 U/mL), amyloglucosidase (from Aspergillus niger, 3330 U/mL), thermostable α‐amylase (from *Bacillus licheniformis* , 3000 U/mL), and glucose oxidase‐peroxidase (GOPOD) reagent were purchased from Megazyme (Wicklow, IRELAND). The other chemicals used were of analytical grade.

### 2.3 Preparation of Samples and Cooking Procedures

For all samples comprising beef, lamb, and chicken liver, raw (control) portions were first set aside for subsequent analyses. Prior to cooking, visible fat, connective tissue, and external membranes were carefully removed from all liver samples to ensure sample uniformity across treatments. The liver samples from each species were then cut into uniform pieces measuring approximately 3 cm × 3 cm × 2 cm, with an average weight of 25 ± 2 g. The same preparation steps were applied to all samples before the different cooking procedures.

Subsequently, four distinct cooking methods—pan‐frying, sous‐vide, boiling, and deep frying—were individually applied to beef, lamb, and chicken liver samples. The equipment used for each cooking method is described in the corresponding subsections. Oil was used only in the pan‐frying and deep‐frying treatments, as required by these cooking procedures, to reflect commonly applied culinary practices and to provide a standardized heat‐transfer medium. To ensure methodological comparability among cooking methods, all treatments were standardized to reach the same endpoint core temperature of 75°C. Differences in oxygen exposure, oil contact, and leaching potential were intentionally treated as intrinsic characteristics of the cooking methods rather than confounding factors. After completion of the cooking process, the samples were cooled to approximately room temperature (25°C), vacuum‐sealed, and stored frozen at −20°C for a maximum of 1 week prior to in vitro digestion and mineral analysis.

#### 2.3.1 Pan‐Frying

The pan‐frying procedure was carried out in a covered stainless steel pan using refined sunflower oil as the cooking medium. Refined sunflower oil was used for all pan‐frying groups to reflect the requirements of this cooking method and to provide a standardized heat‐transfer medium. During the pan‐frying process, the sample‐to‐oil ratio was adjusted to 1:2 (w/v). The pan was preheated to 180°C ± 5°C prior to cooking. Liver samples placed in the pan were pan‐fried for 5 min. During the pan‐frying process, the temperature was monitored using a digital probe thermometer (Portable K‐Thermocouple, Hanna Instruments, Bedfordshire, UK), and cooking was considered complete when the internal temperature of the samples reached 75°C. After cooking, the samples were defatted, and excess surface oil was removed using absorbent paper. They were then allowed to cool at room temperature (25°C ± 1°C).

#### 2.3.2 Boiling

The boiling procedure was carried out in stainless steel pots using deionized water as the cooking medium, without the addition of oil. The sample‐to‐water ratio was set at 1:10 (w/v). The water was heated on a gas stove to 100°C ± 1°C, and once boiling commenced, the liver samples were fully submerged in the water. Samples were boiled for 25 min, with the temperature continuously monitored using a digital thermometer (Portable K‐Thermocouple, Hanna Instruments, Bedfordshire, UK). Cooking was considered complete when the internal temperature of the samples reached 75°C. At the end of the cooking period, samples were removed from the water, drained, and allowed to cool at room temperature (25°C ± 1°C).

#### 2.3.3 Sous‐Vide

After weighing, each liver sample was placed, without the addition of oil, into vacuum‐sealable polyamide/polyethylene bags (bag thickness: 92 μm; temperature resistance: −40°C to +120°C; O_2_ permeability: 9 cm^3^/m^2^ per 24 h at 4°C/80% RH; water vapor permeability: 1.2 g/m^2^ per 24 h) and sealed under 99.6% vacuum using a vacuum packaging machine (Profi Line 40+, Hendi, Robakowo, Poland). The vacuum‐sealed bags were immersed in a thermostatically controlled water bath heated to 75°C (model SW 22, Julabo GmbH, Seelbach, Germany). For sous‐vide cooking, core temperature monitoring was performed using a representative sample in which a thermocouple probe was inserted prior to vacuum sealing. This sample was used exclusively for temperature control purposes, while all analytical samples were cooked under identical time–temperature conditions. The internal temperature of the samples was monitored using a handheld thermometer (Portable K‐Thermocouple, Hanna Instruments, Bedfordshire, UK), and cooking was considered complete when the core temperature reached 75°C. The samples were then held at this temperature for an additional 40 min to ensure thermal uniformity. Immediately after cooking, the vacuum‐sealed bags were removed from the water bath and rapidly cooled in a 2°C ice‐chilled medium for 30 min. Afterward, the samples were equilibrated to room temperature (25°C ± 1°C).

#### 2.3.4 Deep Frying

Refined sunflower oil was used as the cooking medium for the deep‐frying process to reflect the requirements of this cooking method and to provide a standardized heat‐transfer medium. The oil was heated in a stainless steel deep fryer on a gas stove to 180°C ± 5°C. During deep frying, the sample‐to‐oil ratio was adjusted to 1:10 (w/v). Liver samples were deep‐fried for 4 min. During deep frying, the internal temperature of the samples was monitored using a digital probe thermometer (Portable K‐Thermocouple, Hanna Instruments, Bedfordshire, UK), and cooking was considered complete when the internal temperature reached 75°C. Following deep frying, the samples were removed from the oil, excess surface oil was removed using absorbent paper, and the samples were allowed to cool to room temperature (25°C ± 1°C).

### 2.4 Determination of Cooking Loss

The weight reduction before and after thermal processing was used to quantify cooking loss (Woloszyn et al. 2020). Cooking loss was determined to quantify moisture reduction during thermal processing, which is known to influence apparent changes in nutrient and mineral concentrations. Initially, raw liver samples were weighed using an analytical balance. Subsequently, each sample was subjected to thermal processing using four different cooking methods. After completion of the cooking procedures and cooling the samples to room temperature, the cooked samples were reweighed, and cooking loss was determined according to the following formula. These data were later considered in the interpretation of mineral concentration and retention results.

(1) $$\text{Cooking loss}\;\left(\%\right)=\frac{\text{weight before cooking}\;\left(g\right)‐\text{weight after cooking}\;\left(g\right)}{\text{weight before cooking}\;\left(g\right)}\times 100$$

### 2.5 Determination of Retention Coefficients

The extent to which minerals were retained following heat treatment was calculated using the formula provided below (Alfaia et al. 2010). The retention coefficient calculation incorporates both mineral concentration and sample weight before and after cooking, thereby correcting for moisture loss–related concentration effects and allowing the estimation of true mineral retention.

(2) $$R\;\left(\%\right)=\frac{{\mathrm{mc}}_{1}\times w_{1}}{{\mathrm{mc}}_{0}\times w_{0}}\times 100$$

mc_0_: mineral content per gram of liver before cooking, mc_1_: mineral content per gram of liver after cooking, *w*_0_: weight of the liver sample before cooking (g), *w*_1_: weight of the liver sample after cooking (g).

### 2.6 Determination of Mineral Content by **ICP** ‐ **OES**

The mineral content was analyzed using ICP‐OES (iCAP 6500 Duo, Thermo Scientific, Waltham, MA, USA) at the Namık Kemal University Scientific and Technological Research and Application Center (NABİLTEM), following the method described by Vieira et al. (2018), with minor adaptations according to the validated analytical procedure of the accredited laboratory. For mineral determination, approximately 800 mg of homogenized liver sample was accurately weighed and transferred into microwave digestion vessels. The samples were digested using a mixture of 3 mL of HNO_3_, 1 mL of H_2_O_2_, and 2 mL of deionized water, corresponding to an HNO_3_:H_2_O_2_:H_2_O ratio of 3:1:2 (v/v/v). Nitric acid 65% and hydrogen peroxide 30% were used for sample digestion. After microwave‐assisted digestion, the digests were allowed to cool to room temperature, transferred to polypropylene flasks, filtered when necessary, and diluted to a final volume of 30 mL with deionized water prior to ICP‐OES analysis. This procedure was repeated three times for each sample. The minerals Na, Mg, K, P, Fe, Cu, Mn, Zn Co, and Cr were determined by ICP‐OES, and their concentrations were calculated using external calibration curves prepared from certified multi‐element standards. Calibration for mineral determination was performed using certified multi‐element analytical solutions prepared from 1000 mg L^−1^ stock standard solutions (SpecSol, São Paulo, Brazil) diluted in 1% (v/v) HNO_3_ medium according to the validated analytical procedure of the accredited laboratory (NABİLTEM). Mineral concentrations were reported on a wet‐weight basis, since no additional drying step was applied prior to digestion, and the sample mass used for calculation corresponded to the cooked, undried material. The operating parameters of the ICP‐OES were as follows: RF applied power 1.15 kW, Plasma gas flow rate 12 L min^−1^, Auxiliary gas flow rate 0.5 L min^−1^, Nebulizer gas flow 0.7 L min^−1^, Nebulizer Concentric, Spray chamber Cyclonic, Operating mode Axial.

The LOD and LOQ values were obtained from the analytical reports provided by the accredited laboratory (NABİLTEM) based on the ICP‐OES instrument calibration and validation procedures.

LOD: limit of detection; LOQ: limit of quantification; Na: LOD = 0.034 mg/L; LOQ = 0.113333 mg/L; Mg: LOD = 0.00012 mg/L; LOQ = 0.0004 mg/L; K: LOD = 0.0411 mg/L; LOQ = 0.137 mg/L; P: LOD = 0.0088 mg/L; LOQ = 0.029333 mg/L; Fe: LOD = 0.002108 mg/L; LOQ = 0.007027 mg/L; Cu: LOD = 1.384 ppb = 0.001384 mg/L; LOQ = 0.004613 mg/L; Mn: LOD = 0.000305 mg/L; LOQ = 0.001017 mg/L; Zn: LOD = 0.001071 mg/L; LOQ = 0.003570 mg/L; Co: LOD = 0.002492 mg/L; LOQ = 0.008307 mg/L; Cr: LOD = 0.007506 mg/L; LOQ = 0.02502 mg/L.

### 2.7 In Vitro Digestion Procedure and Determination of Bioaccessibility

Simulated in vitro digestion was performed based on the standardized protocol described by Minekus et al. (2014), with minor modifications. The digestion process was carried out sequentially to include the oral, gastric, and intestinal phases, and samples were collected at the end of each phase. During the oral phase, 0.5 g of each sample was mixed with simulated salivary fluid and CaCl_2_ (0.3 M). The total volume was adjusted with distilled water, and the mixture was incubated in a shaking water bath at 37°C for 2 min. In the gastric phase, simulated gastric fluid and CaCl_2_ (0.3 M) were added to each sample, the pH was adjusted to 3 using 1 M HCl, and pepsin was subsequently added. The volume was brought to the desired level with distilled water, and the mixture was incubated at 37°C in a shaking water bath for 2 h. For the intestinal phase, simulated intestinal fluid and CaCl_2_ (0.3 M) were added, the pH was adjusted to 7 using 1 M NaOH, followed by the addition of pancreatin and bile. The total volume was adjusted with distilled water, and the mixture was incubated at 37°C in a shaking water bath for 2 h. Mineral bioaccessibility was evaluated using the soluble fraction obtained after in vitro digestion. Following the gastric and intestinal digestion phases, the digested samples were centrifuged at 5000 rpm for 20 min at 4°C to separate the soluble (bioaccessible) fraction. Subsequently, 2 mL of the resulting supernatant was collected for mineral analysis and stored at −20°C for a maximum of 1 week until ICP‐OES determination.

Mineral bioaccessibility was expressed as a Bioaccessibility Index (BI%), calculated as the ratio of mineral concentration measured in the intestinal soluble fraction to the total mineral concentration of the corresponding cooked sample. BI values exceeding 100% were interpreted as an increased release of minerals from the food matrix during in vitro digestion, reflecting matrix disruption and solubilization effects rather than absolute intestinal absorption potential.

The Bioaccessibility Index (BI%) was calculated to describe the relative release of minerals into the soluble intestinal fraction during digestion, according to Equation (3):

(3) $$\mathrm{BI}\%=\frac{\text{Post}‐\text{digestion mineral concentration}}{\text{Total mineral concentration}\;\left(\text{Cooked sample}\right)}\times 100$$

Mineral bioaccessibility (%) was calculated as the proportion of mineral released into the soluble fraction after in vitro digestion relative to the mineral content of the cooked sample prior to digestion.

### 2.8 Statistical Analysis

All experiments were performed in triplicate for each liver type and cooking method. Each digested sample was measured three times by ICP‐OES, and the instrumental readings were averaged before statistical analysis. Results are expressed as mean ± standard deviation based on three independent analytical replicates (*n* = 3).

Statistical analyses were performed using IBM SPSS Statistics, version 22 (IBM Corp., New York, USA). One‐way analysis of variance (ANOVA) was applied for pre‐planned comparisons among groups. Specifically, cooking methods were compared within each liver type, and liver types were compared within the same cooking method, as indicated in the table footnotes. When ANOVA indicated statistically significant differences, Tukey's HSD post hoc test was used to identify pairwise differences among groups. The 10 minerals analyzed in the study were evaluated as separate outcome variables and were not considered as statistical replicates. Statistical significance was set at *p* < 0.05.

## 3 Results and Discussion

### 3.1 Effect of Different Cooking Methods on Cooking Losses in Liver Samples

Cooking loss, defined as the combined loss of moisture and water‐soluble components during thermal processing (Chumngoen et al. 2016), is a critical parameter that influences juiciness, product yield, and economic value. Table 1 summarizes the cooking loss percentages for beef, lamb, and chicken liver samples subjected to different cooking methods. In all liver types, cooking losses varied significantly depending on the applied thermal treatment (*p* < 0.05). However, when different liver types were compared within the same cooking method, no statistically significant differences were observed among beef, lamb, and chicken liver samples (*p* > 0.05).

**TABLE 1:** Cooking losses in liver samples (pan‐frying, sous‐vide, boiling, deep frying).

Sample	Cooking loss (%)	Sample	Cooking loss (%)	Sample	Cooking loss (%)
BP	28.10 ± 1.65^aA^	LP	27.42 ± 1.20^aA^	CP	26.67 ± 1.45^aA^
BV	21.95 ± 0.74^bB^	LV	22.79 ± 0.68^bB^	CV	20.94 ± 0.86^bB^
BB	25.64 ± 1.14^cC^	LB	25.39 ± 1.13^cC^	CB	23.21 ± 0.56^cC^
BF	32.26 ± 1.38^dD^	LF	29.72 ± 1.00^dD^	CF	29.00 ± 1.49^dD^

*Note:* Different lowercase letters indicate significant differences among cooking methods within the same liver type, whereas different uppercase letters indicate significant differences among liver types within the same cooking method (*p* < 0.05).

Abbreviations: BB, beef boiling; BF, beef deep frying; BP, beef pan‐frying; BV, beef sous‐vide; CB, chicken boiling; CF, chicken deep frying; CP, chicken pan‐frying; CV, chicken sous‐vide; LB, lamb boiling; LF, lamb deep frying; LP, lamb pan‐frying; LV, lamb sous‐vide.

Cooking losses during boiling originate from the shrinkage of myofibrils, starting at approximately 40°C; as the temperature rises, the reduction in inter‐fibrillar space further diminishes the water‐holding capacity of the tissue. At 56°C–62°C, the contraction of the perimysial connective tissue surrounding the muscle fiber bundles facilitates the release of water. Moreover, prolonged cooking time enhances the contraction of connective tissue and the toughening of myofibrils (Chumngoen et al. 2016; Oz et al. 2017). The sous‐vide method resulted in the lowest cooking loss across all liver types, with values ranging from 20.94 ± 0.86 to 22.79 ± 0.68 (*p* < 0.05). This difference was attributed to the temperature variations among the applied treatments. The sous‐vide technique applies a lower cooking temperature compared to other methods. Furthermore, in the sous‐vide method, the liver is heated within a plastic pouch, which prevents the evaporation of its moisture into the air, representing an additional factor contributing to the lower cooking loss. The extent of cooking loss is dependent on the meat temperature and the duration of the applied thermal treatment (Palka and Daun 1999; Sánchez del Pulgar et al. 2012). Deep frying, which involved the highest temperature treatment, resulted in the most significant cooking loss (29.00 ± 1.49–32.26 ± 1.38). The lower cooking loss observed in sous‐vide samples is consistent with previous studies indicating that controlled low‐temperature sous‐vide cooking can reduce water loss and better preserve moisture in meat products, although this effect may vary depending on time–temperature conditions (Sánchez del Pulgar et al. 2012; Li 2025). In contrast, the higher cooking loss observed after deep frying may be associated with high processing temperature and increased moisture evaporation during frying. This interpretation is supported by Alugwu et al. (2022), who reported that deep‐fat frying increased cooking loss in chicken breast meat, particularly at higher frying temperatures and longer cooking times. In addition, heating‐induced protein denaturation, myofibrillar shrinkage, and reduced water‐holding capacity may further contribute to cooking loss in meat systems (Palka and Daun 1999; Tornberg 2005). Although cooking loss largely reflects moisture loss, this may lead to apparent increases in mineral concentrations on a weight basis. To avoid interpreting such concentration effects as actual mineral gains, true retention coefficients were calculated to assess mineral retention more accurately.

### 3.2 Effects of Cooking Methods and Liver Type on Mineral Content

In this study, the concentrations of sodium (Na), magnesium (Mg), potassium (K), phosphorus (P), iron (Fe), copper (Cu), manganese (Mn), zinc (Zn), chromium (Cr), and cobalt (Co) were determined in liver samples from three different species (beef, lamb, and chicken), either raw or subjected to four distinct cooking methods (pan‐frying, sous‐vide, boiling, and deep frying), and the results are presented in Table 2. The mineral content of raw and cooked liver samples showed significant differences in the planned one‐way ANOVA comparisons, both among cooking methods within each liver type and among liver types within the same cooking method (*p* < 0.05). All mineral concentrations were determined following microwave digestion of homogenized samples and are expressed on a mass basis (mg/kg). Because thermal processing induces moisture loss, apparent increases in mineral concentrations in cooked samples may partially reflect matrix concentration effects rather than true mineral retention. Therefore, mineral concentration data were interpreted in conjunction with cooking loss measurements and true retention coefficients, which account for sample weight reduction and provide a more accurate assessment of actual mineral preservation during thermal processing.

**TABLE 2:** Mineral content (mg/kg) of raw and cooked (pan‐frying, sous‐vide, boiled, and deep frying) beef, lamb, and chicken liver samples.

Sample	Na	Mg	K	P	Fe	Cu	Mn	Zn	Cr	Co
BR	1383.5 ± 86.13^aA^	151.36 ± 4.89^aA^	5663.84 ± 259.96^aA^	576.90 ± 19.53^aA^	113.42 ± 6.99^aA^	175.37 ± 6.44^aA^	6.07 ± 0.27^aA^	94.95 ± 3.87^aA^	nd	nd
BP	1125.50 ± 45.40^bA^	117.64 ± 4.58^bA^	4976.34 ± 172.87^bA^	371.24 ± 18.50^bA^	119.61 ± 6.70^aA^	182.35 ± 7.82^aA^	5.04 ± 0.25^bA^	88.87 ± 2.75^aA^	nd	nd
BV	1407.77 ± 74.74^aA^	196.49 ± 9.44^cA^	5732.80 ± 307.78^aA^	582.97 ± 27.14^aA^	181.17 ± 10.88^bA^	201.31 ± 10.25^bA^	6.91 ± 0.31^cA^	124.33 ± 9.22^bA^	nd	nd
BB	578.74 ± 34.08c^A^	62.34 ± 2.40^dA^	2277.47 ± 99.50^cA^	229.85 ± 15.10^cA^	188.76 ± 2.77^bA^	183.07 ± 11.23^aA^	4.60 ± 0.25^bA^	98.15 ± 4.74^aA^	nd	nd
BF	1130.73 ± 69.81^bA^	94.34 ± 6.55^eA^	4602.16 ± 297.45^bA^	458.22 ± 24.61^dA^	189.88 ± 8.06^bA^	173.88 ± 3.06^aA^	5.80 ± 0.25^aA^	91.72 ± 3.72^aA^	nd	nd
LR	1145.61 ± 41.44^aB^	181.25 ± 5.72^aB^	5360.28 ± 129.96^aA^	517.25 ± 17.83^aB^	143.77 ± 5.36^aB^	751.80 ± 27.71^aB^	9.46 ± 0.35^aB^	96.76 ± 2.80^aA^	nd	nd
LP	1088.91 ± 14.42^bA^	189.26 ± 6.53^bB^	5063.43 ± 144.10^bA^	500.25 ± 18.80^aB^	92.66 ± 3.55^bB^	664.86 ± 20.36^bB^	8.40 ± 0.22^bB^	78.06 ± 1.61^bB^	nd	nd
LV	1310.90 ± 35.25^cA^	198.59 ± 4.21^cA^	5467.47 ± 89.78^aA^	538.85 ± 19.36^bB^	156.85 ± 5.07^cB^	803.93 ± 20.01^cB^	10.62 ± 0.42^cB^	97.64 ± 2.73^aB^	nd	nd
LB	659.14 ± 18.050^dB^	53.13 ± 1.46d^B^	2450.94 ± 78.42^cB^	265.20 ± 9.52^cB^	181.56 ± 3.86^dA^	849.03 ± 21.72^dB^	8.58 ± 0.28^bB^	101.22 ± 3.28^cA^	nd	nd
LF	1106.86 ± 32.24^bA^	92.62 ± 2.68^eA^	4595.83 ± 119.97^dA^	460.78 ± 15.30^dA^	187.54 ± 2.99^eA^	560.05 ± 16.81^eA^	8.68 ± 0.23^bB^	89.15 ± 2.52^dA^	nd	nd
CR	1825.91 ± 83.21^aC^	357.52 ± 11.34^aC^	6122.32 ± 378.13^aB^	620.13 ± 26.45^aC^	214.75 ± 11.67^aC^	14.68 ± 0.72^aC^	8.06 ± 0.60^aC^	108.08 ± 3.26^aB^	nd	nd
CP	1745.47 ± 80.34^abB^	293.68 ± 12.41^bC^	5967.59 ± 417.23^aB^	601.06 ± 35.36^aC^	182.67 ± 7.83^bC^	18.43 ± 0.69^bC^	4.34 ± 0.17^bC^	153.51 ± 9.08^bC^	nd	nd
CV	1879.51 ± 129.43^acB^	277.99 ± 4.97^cB^	6201.33 ± 317.66^aA^	637.14 ± 26.12^aC^	281.6 ± 7.86^cC^	16.67 ± 0.82^cC^	8.58 ± 0.51^cC^	136.28 ± 6.99^cC^	nd	nd
CB	825.34 ± 39.63^dC^	181.45 ± 6.40^dC^	2455.13 ± 86.19^bB^	265.71 ± 14.30^bB^	204.22 ± 11.34a^dB^	22.52 ± 1.14^dC^	7.14 ± 0.35^dC^	168.39 ± 5.30^dB^	nd	nd
CF	1578.90 ± 69.61^eB^	295.06 ± 9.05^bB^	5534.80 ± 180.43^cB^	541.49 ± 35.07^cB^	226.38 ± 11.24^aeB^	21.80 ± 1.13^dC^	6.30 ± 0.29^eC^	113.09 ± 2.30^aB^	nd	nd

*Note:* Lowercase letters indicate differences in mineral content between raw and cooked samples within the same liver type, with different letters denoting statistical significance at *p* < 0.05. Uppercase letters indicate differences in mineral content between raw and cooked samples of different liver types prepared by the same cooking method, with different letters denoting statistical significance *p* < 0.05.

Abbreviations: BB, beef boiling; BF, beef deep frying; BP, beef pan‐frying; BR, beef raw; BV, beef sous‐vide; CB, chicken boiling; CF, chicken deep frying; CP, chicken pan‐frying; CR, chicken raw; CV, chicken sous‐vide; LB, lamb boiling; LF, lamb deep frying; LP, lamb pan‐frying; LR, lamb raw; LV, lamb sous‐vide; nd, not detected.

Previous studies have shown that liver has a distinct mineral profile compared with skeletal muscle. Tomović et al. (2011) reported that P, Na, Ca, Zn, Fe, Cu, and Mn contents were significantly higher in pig liver than in semimembranosus muscle, highlighting the mineral density of liver tissue. Similarly, Latoch et al. (2024) emphasized that edible offal, especially liver, represents a nutrient‐dense food matrix rich in essential micronutrients, including Fe, Cu, P, and Zn. Studies on offal from calves and ruminants also indicate that mineral composition varies according to organ type and animal species (Biel et al. 2019; Florek et al. 2012). Although direct studies on cooked beef, lamb, and chicken liver are limited, comparisons with cooked skeletal muscle products provide useful mechanistic context for interpreting cooking‐induced mineral changes, particularly leaching of water‐soluble minerals into the cooking medium, moisture loss, protein denaturation, and matrix concentration effects.

Thermal processing notably alters the nutritional profile of liver by improving nutrient bioaccessibility and matrix disintegration (Białobrzewski et al. 2010), while potentially reducing mineral retention as a result of leaching into cooking media and redistribution within the food matrix. Pan‐frying, boiling, and deep frying resulted in significant reductions (*p* < 0.05) in mineral concentrations compared with raw samples. Boiling and deep frying treatments caused statistically significant decreases in all macrominerals (Na, Mg, K, P). The cooking process facilitates the leaching of inorganic compounds, such as Na, K, Ca, Mg, and P, from foods into water (Wereńska et al. 2023). Boiling resulted in a pronounced reduction in the content of water‐soluble minerals (Na, Mg, K, and P). In the BB sample, macro‐mineral reductions ranged from 58.17% to 60.14%, while CB showed similar reductions (54.78%–62.13%). In LB, reductions varied from 42.45% to 70.68%, with Mg exhibiting the most significant decrease, from 181.25 ± 5.72 mg/kg to 53.13 ± 1.46 mg/kg (70.68%). Wereńska et al. (2023) reported that boiling goose meat increased P (from 603.80 ± 37.58 to 1038.73 ± 12.94 mg/100 g) and Mg (from 74.10 ± 2.80 to 114.57 ± 1.97 mg/100 g), while Na (from 311.18 ± 55.27 mg/100 g to 152.15 ± 6.05 mg/100 g) and K (from 1148.97 ± 334.46 mg/100 g to 837.69 ± 131.61 mg/100 g) decreased. During boiling, the reduction in mineral content is attributed to the leaching of water‐soluble minerals into the cooking medium. Conversely, increases observed for certain trace elements following boiling should be interpreted cautiously, as they may partly result from moisture reduction rather than absolute mineral gains, a distinction clarified through retention coefficient analysis. Boiling generally increased trace element concentrations, except for Mn, which decreased significantly in all liver types (*p* < 0.05). Fe content rose significantly in beef and lamb (*p* < 0.05) but slightly reduced in chicken (*p* > 0.05). Boiling also enhanced Cu and Zn levels in all samples, with the highest Fe increase in beef liver (66.43%) and the highest Cu and Zn increases in chicken liver, respectively 53.41% and 55.8%. Wereńska et al. (2023) reported that boiling goose meat increased zinc from 3.70 ± 0.19 to 9.20 ± 0.23 mg/100 g and iron from 14.32 ± 2.16 to 24.42 ± 0.61 mg/100 g. Da Silva et al. (2017) observed decreases in copper (282.6 ± 3.2 to 183.5 ± 17.2 mg/kg), iron (116.5 ± 5.7 to 91.1 ± 13.0 mg/kg), and zinc (131.5 ± 1.1 to 111.9 ± 7.5 mg/kg) during boiling.

Deep frying led to notable reductions in macromineral levels compared to raw samples, although these reductions were less pronounced than those observed after boiling. In beef and lamb livers, deep frying did not cause statistically significant differences in mineral contents, except for Mn. In contrast, chicken liver exhibited a considerable variation compared to the other species (*p* < 0.05). Upon deep frying, beef liver exhibited a 37.66% reduction in Mg, while the decreases observed in other minerals ranged from 18% to 21%. In LF samples, Mg decreased by nearly 50% compared to the raw state. This decline may be associated with solubility‐ and diffusion‐driven release, as well as disruption of intracellular distribution or potential migration into the extracellular matrix. Sodium showed minimal reduction during deep frying, with only a 3.38% decrease, indicating relative stability under the applied cooking conditions. In chicken liver, the reduction in macrominerals ranged from 10% to 17%, with potassium exhibiting the lowest decrease (9.59%). Following deep frying, Fe levels increased in all liver types, reaching statistically significant levels in beef and lamb samples (*p* < 0.05). Although the CF samples showed the highest Fe level (226.38 ± 11.24 mg/kg), the BF group exhibited the most significant relative increase, reaching 189.88 ± 8.06 mg/kg (+67.39%). These apparent increases are consistent with the combined effects of moisture loss and thermal concentration, emphasizing the relevance of retention‐based evaluation when interpreting deep frying‐induced changes. Among the deep fried samples, Cu exhibited a significant increase only in chicken liver, reaching 21.8 ± 1.13 mg/kg. In contrast, Mn levels decreased across all liver types, with statistically significant reductions observed in lamb and chicken samples (*p* < 0.05). At high temperatures, such as during frying, proteins form a crust on the surface of the meat, thereby reducing the leaching of soluble minerals (Yong et al. 2019).

Pan‐frying significantly affected the mineral composition of all liver types (*p* < 0.05). In BS samples, all macrominerals decreased, with phosphorus showing the highest reduction, exceeding 35% (*p* < 0.05). Lamb and chicken livers also exhibited decreases in macromineral contents, although the reductions were less pronounced than in beef liver. Notably, only LS samples showed a significant increase in Mg content, rising from 181.25 ± 5.72 to 189.26 ± 6.53 mg/kg (*p* < 0.05). Overall, pan‐frying exerted a reducing effect on trace minerals; all trace minerals in LP samples declined significantly (*p* < 0.05). Mn content decreased substantially across all pan‐fried samples, consistent with the trends observed following boiling and frying (*p* < 0.05). Significant increases following pan‐frying were observed only in the CS sample, with Cu rising from 14.68 ± 0.72 to 18.43 ± 0.69 mg/kg and Zn from 108.08 ± 3.26 to 153.51 ± 9.08 mg/kg. During pan‐frying, high temperature and direct contact with the heated cooking surface may promote tissue disruption and moisture loss, facilitating the release of soluble minerals into cooking exudates; however, simultaneous moisture reduction may concentrate certain minerals per unit weight. Accordingly, concentration‐based results were evaluated in conjunction with cooking loss and retention coefficients to distinguish true mineral preservation from dehydration‐driven effects. Additionally, cooking in a lipid‐rich medium can affect mineral stability and distribution within the tissue matrix, contributing to both preservation and redistribution across cellular compartments.

Sous‐vide proved to be the most effective method for mineral preservation. Except for potassium (K), mineral contents differed significantly among liver types subjected to sous‐vide (*p* < 0.05). In beef liver, sous‐vide cooking resulted in increases in all macro minerals, with Mg exhibiting a notable rise of 29.82%. In lamb liver, significant enhancements were observed, particularly in Na, Mg, and P levels (*p* < 0.05). During sous‐vide cooking, the absence of direct contact between the food and water, combined with the presence of only the water naturally contained within the food and the application of controlled thermal gradients, minimizes mineral leaching and thereby prevents nutrient losses. Prolonged cooking at low temperatures enhances mineral stability (Da Silva et al. 2017; Wereńska et al. 2023). Furthermore, vacuum sealing and hermetic packaging may limit the loss of water‐soluble minerals by reducing their transfer into the surrounding cooking medium during sous‐vide processing. Although sous‐vide cooking led to increases in Na, K, and P levels in chicken liver, these changes were not statistically significant (*p* > 0.05). In contrast, Mg content decreased significantly by 21.58% (*p* < 0.05), suggesting a selective reduction of Mg in chicken liver under sous‐vide conditions. This effect may be associated with the distribution and solubility characteristics of Mg within the tissue rather than a general effect of vacuum processing. In contrast, sous‐vide treatment significantly enhanced trace mineral levels (Fe, Cu, Mn, and Zn) in all BV, LV, and CV samples compared to raw tissues (*p* < 0.05). Notably, the high retention coefficients observed for sous‐vide–treated samples indicate that these increases largely reflect genuine mineral preservation rather than concentration effects associated with moisture loss. The highest Fe concentration was recorded in CV (281.6 ± 7.86 mg/kg), while LV showed the second highest Cu level (803.93 ± 20.01 mg/kg). Macharáčková et al. (2021) reported that sous‐vide cooking of pork decreased sodium from 487.99 ± 44.73 to 412.93 ± 44.98 mg/kg and potassium from 4502.00 ± 208.14 to 4070.06 ± 310.07 mg/kg, while magnesium remained stable (354.77 ± 31.58 to 353.70 ± 23.44 mg/kg). Furthermore, iron levels decreased from 8.48 ± 1.09 to 6.25 ± 1.02 mg/kg, whereas copper and zinc levels increased from 0.48 ± 0.11 to 0.63 ± 0.12 mg/kg and from 19.29 ± 2.78 to 22.66 ± 3.36 mg/kg, respectively. Falowo et al. (2017) reported that sous‐vide cooking of beef liver decreased sodium from 43.90 to 32.90 mg/100 g, while magnesium, potassium, and zinc increased from 41.80 to 49.80 mg/100 g, 171.8 to 248.27 mg/100 g, and 8.50 to 12.78 mg/100 g, respectively.

### 3.3 Effect of Cooking and Liver Type on Mineral Retention

The percentage retention values of beef, lamb, and chicken livers following cooking (pan‐frying, sous‐vide, boiling, and deep frying) are presented in Table 3. The percentage retention values of minerals in beef, lamb, and chicken livers differed significantly among the various cooking methods (*p* < 0.05). Only after sous‐vide cooking did the levels of K, P, and Mn not show statistically significant differences among the different liver types (*p* > 0.05). The use of true retention coefficients, incorporating both concentration changes and cooking‐induced weight loss, enabled a more accurate assessment of actual mineral preservation rather than apparent increases driven by moisture loss.

**TABLE 3:** Mineral retention values (%) in beef, lamb, and chicken liver samples cooked using different methods (pan‐frying, sous‐vide, boiling, deep frying).

Sample	Na	Mg	K	P	Fe	Cu	Mn	Zn	Cr	Co
BP	58.75 ± 5.32^aA^	56.00 ± 4.25^aA^	63.31 ± 4.30^aA^	46.29 ± 1.68^aA^	76.25 ± 8.53^aA^	74.9 ± 5.40^aA^	59.8 ± 4.35^aA^	67.42 ± 4.42^aA^	nd	nd
BV	79.71 ± 6.38^bA^	101.45 ± 6.21^bA^	79.18 ± 5.81^bx^	78.97 ± 3.67^bx^	125.15 ± 11.20^bA^	89.81 ± 7.07^bA^	88.97 ± 5.09^bx^	102.37 ± 8.54^bA^	nd	nd
BB	31.21 ± 2.54^cA^	30.65 ± 1.34^cA^	29.94 ± 1.64^cA^	29.63 ± 1.45^cA^	124.23 ± 8.74^bA^	77.76 ± 5.93^aA^	56.41 ± 4.84^acA^	77.01 ± 5.32^cA^	nd	nd
BF	55.62 ± 5.40^aA^	42.25 ± 2.92^dA^	55.13 ± 4.08^dA^	53.86 ± 2.79^dA^	113.76 ± 8.05^bA^	67.24 ± 2.66^cA^	64.76 ± 2.94^adA^	65.54 ± 4.05^aA^	nd	nd
LP	69.05 ± 2.30^aB^	75.81 ± 2.70^aB^	68.58 ± 2.49^aAB^	70.25 ± 3.32^aB^	46.83 ± 2.43^aB^	64.23 ± 2.36^aB^	64.55 ± 3.59^aB^	58.57 ± 1.58^aB^	nd	nd
LV	88.41 ± 2.84^bB^	84.64 ± 2.51^bB^	78.77 ± 1.55^bx^	80.49 ± 3.31^bx^	84.35 ± 4.11^bB^	82.66 ± 3.41^bB^	86.75 ± 2.63^bx^	77.99 ± 3.63^bB^	nd	nd
LB	42.94 ± 0.94^cB^	21.90 ± 1.22^cB^	34.12 ± 1.36^cB^	38.29 ± 1.86^cB^	94.32 ± 3.68^cB^	84.38 ± 4.27^bA^	67.78 ± 3.77^aB^	78.11 ± 3.54^bA^	nd	nd
LF	67.97 ± 2.86^aB^	35.95 ± 1.54^dB^	60.3 ± 2.46^dB^	62.67 ± 2.76^dB^	91.8 ± 3.77^cB^	52.44 ± 2.84^cB^	64.57 ± 3.10^aA^	64.81 ± 2.84^cA^	nd	nd
CP	70.22 ± 4.67^aB^	60.30 ± 3.69^aC^	71.71 ± 6.80^aB^	71.11 ± 4.04^aB^	62.57 ± 5.05^aC^	92.17 ± 4.73^aC^	39.69 ± 2.84^aC^	104.25 ± 7.67^aC^	nd	nd
CV	81.6 ± 7.34^bAB^	61.52 ± 1.83^aC^	80.46 ± 7.40^bx^	81.29 ± 3.19^bx^	141.05 ± 9.93^bC^	89.95 ± 6.20^aA^	84.75 ± 8.57^bx^	99.86 ± 7.11^aA^	nd	nd
CB	34.79 ± 2.39^cC^	39.00 ± 1.63^bC^	30.95 ± 2.71^cA^	32.96 ± 2.23^cC^	73.24 ± 5.57^cC^	118.13 ± 9.48^bB^	68.34 ± 5.48^cB^	119.78 ± 5.62^bB^	nd	nd
CF	61.50 ± 3.80^dC^	58.61 ± 1.92^aC^	64.39 ± 4.30^dB^	61.97 ± 2.58^dB^	75.06 ± 5.84^cC^	105.55 ± 6.11^cC^	55.88 ± 5.73^dB^	74.32 ± 1.90^cB^	nd	nd

*Note:* Lowercase letters indicate statistically significant differences (*p* < 0.05) in mineral retention percentages among cooking methods within the same liver type. Uppercase letters indicate statistically significant differences (p<0.05) in mineral retention percentages among liver types within the same cooking method. X indicates that no statistically significant difference was found the compared samples (*p* > 0.05).

Abbreviations: BB, beef boiling; BF, beef deep frying; BP, beef pan‐frying; BV, beef sous‐vide; CB, chicken boiling; CF, chicken deep frying; CP, chicken pan‐frying; CV, chicken sous‐vide; LB, lamb boiling; LF, lamb deep frying; LP, lamb pan‐frying; LV, lamb sous‐vide; nd, not detected.

The retention of minerals and other nutrients in foods subjected to thermal processing is crucial for maintaining a balanced diet (Goluch et al. 2021). Boiling resulted in the lowest macromineral retention across all liver types, with beef liver retaining significantly less Na and K than lamb and chicken, indicating higher susceptibility of bovine tissues to mineral leaching. In pan‐fried samples, LP and CP showed significantly different retention compared to BS but were similar to each other. Sous‐vide cooking resulted in the highest macromineral retention across all liver types, with the maximum value observed for magnesium in BV (101.45 ± 6.21). Since the sous‐vide cooking process does not require water, it likely allows for a higher retention of minerals compared to other cooking methods (Wereńska et al. 2023). Modzelewska‐Kapituła et al. (2019) reported that Na, Mg, and K retention in beef were 58.7%, 69.3%, and 57.8% after steam cooking, and 64.4%, 70.4%, and 63.5% after sous‐vide cooking. Wereńska et al. (2023) reported retention values in goose meat after sous‐vide cooking of 36.59%, 93.43%, 60.27%, and 117.05% for Na, Mg, K, and P, respectively. In contrast, after boiling, the corresponding retention values were 28.70%, 83.89%, 40.52%, and 92.86%. Macharáčková et al. (2021) reported that following sous‐vide cooking of pork, the retention of Na, Mg, and K was 59.79%, 66.22%, and 56.28%, respectively.

Trace minerals exhibited greater stability compared to macro minerals. Macro minerals such as K and Na, which are present in meat predominantly in relatively simple inorganic forms, have a higher likelihood of being released into the cooking medium compared to minerals like Fe, Cu, and Zn that are more tightly associated with proteins (Macharáčková et al. 2021). Iron exhibited the highest retention overall, with the CV sample reaching a value of 141.05 ± 9.93. The highest Mn retention occurred with sous‐vide, while Cu and Zn reached their peaks after boiling (118.13 ± 9.48 and 119.78 ± 5.62, respectively). Retention values exceeding 100% may be attributed to analytical variability and matrix changes during cooking, as previously reported in the literature. When evaluated in terms of liver type, it is observed that the micromineral retention values of chicken samples are generally higher than those of the other types. Modzelewska‐Kapituła et al. (2019) reported that steam‐cooked beef retained Fe, Cu, Mn, and Zn at 92.9%, 101.4%, 64.0%, and 110.6%, respectively, whereas sous‐vide treatment yielded 62.4%, 86.5%, 60.0%, and 97.9% retention. Wereńska et al. (2023) reported Fe retention of 89.13% (sous‐vide) and 92.89% (boiling) and Zn retention of 140.03% and 142.70%, respectively, in goose meat. Macharáčková et al. (2021) reported that, following sous‐vide cooking of pork, the retention of Fe, Cu, and Zn was 51.98%, 75.33%, and 82.56%, respectively.

### 3.4 Mineral Contents of Liver Samples Subjected to Different Cooking Methods Following In Vitro Gastric and Intestinal Digestion

The mineral contents of liver samples (beef, lamb, and chicken) subjected to different cooking methods (pan‐frying, sous‐vide, boiling, and deep frying) followed by in vitro digestion are presented in Table 4. In almost all samples, statistically significant differences were observed between post‐cooking mineral values and mineral values in the gastric and intestinal environments after in vitro digestion (except for Mn in LF, Zn in BB, and Zn in CP) (*p* < 0.05). All mineral concentrations are expressed on a wet‐weight basis; therefore, changes observed after cooking and digestion may partly reflect moisture loss–induced concentration effects rather than true mineral preservation. To distinguish apparent concentration increases from actual mineral retention, cooking loss and true retention coefficients were evaluated separately. The use of true retention coefficients accounts for both concentration changes and sample weight reduction, allowing a more accurate assessment of mineral preservation following thermal processing. It should be noted that mineral concentrations reported after in vitro digestion represent the soluble fraction released into the digestion medium and thus reflect matrix disintegration and dilution effects associated with simulated gastrointestinal conditions, rather than the absolute mineral content of the original food matrix.

**TABLE 4:** Mineral contents (mg/kg) in the bioaccessible (soluble) fraction of beef, lamb, and chicken liver samples cooked by different methods (pan‐frying, sous‐vide, boiling, and deep frying) after in vitro gastric and intestinal digestion.

Sample	Na	Mg	K	P	Fe	Cu	Mn	Zn	Cr	Co
*Beef*										
BP	1125.50 ± 45.40^a^	117.64 ± 4.58^a^	4976.34 ± 172.87^a^	371.24 ± 18.50^a^	119.61 ± 6.70^a^	182.35 ± 7.82^a^	5.04 ± 0.25^a^	88.87 ± 2.75^a^	nd	nd
BPS	71046.40 ± 48.90b^AI^	1464.40 ± 5.54^bAI^	8153.00 ± 165.80^bAI^	2174.40 ± 19.07^bAI^	344.60 ± 7.80^bAI^	40.64 ± 1.78^bAI^	8.05 ± 0.03^bAI^	84.64 ± 0.28^bAI^	nd	nd
BPI	186.01 ± 34.14^cE^L	3880.00 ± 318.00^cEL^	23164.00 ± 806.92^cEL^	11330.00 ± 433.73^cEL^	142.40 ± 7.09^cEL^	155.10 ± 9.74^cEL^	12.92 ± 0.28^cEL^	92.39 ± 1.91^cEL^	nd	nd
BV	1407.77 ± 74.74^a^	196.49 ± 9.44^a^	5732.80 ± 307.78^a^	582.97 ± 27.14^a^	181.17 ± 10.88^a^	201.31 ± 10.25^a^	6.91 ± 0.31^a^	124.33 ± 9.22^a^	nd	nd
BVS	76674.80 ± 940.58^bBI^	1161.60 ± 57.93^bBI^	6900.80 ± 126.35^bBI^	2475.60 ± 137.10^bBI^	647.66 ± 13.88^bBI^	33.60 ± 5.26^bBI^	4.40 ± 0.13^bBI^	81.21 ± 2.38^bBI^	nd	nd
BVI	131.14 ± 20.03^cFX^	2967.00 ± 309.95^cFL^	17,758.00 ± 596.30^cFL^	9813.00 ± 477.48^cFL^	188.10 ± 20.85^cFL^	135.04 ± 19.80^cFL^	11.31 ± 0.38^cFL^	74.88 ± 1.48^cFL^	nd	nd
BB	578.74 ± 34.08^a^	62.34 ± 2.40^a^	2277.47 ± 99.50^a^	229.85 ± 15.10^a^	188.76 ± 2.77^a^	183.07 ± 11.23^a^	4.60 ± 0.25^a^	98.15 ± 4.74^a^	nd	nd
BBS	73910.40 ± 712.30^bCI^	668.80 ± 65.96^bCI^	3942.00 ± 136.88^bCI^	1282.80 ± 77.47^bCI^	842.40 ± 72.28^bCI^	28.40 ± 3.12^bCI^	4.89 ± 0.10^bCI^	81.50 ± 3.55^bBI^	nd	nd
BBI	139.43 ± 16.75^aFL^	2647.00 ± 210.43^cGL^	15,882.00 ± 525.50^cGL^	7590.00 ± 390.97^cGL^	437.14 ± 23.16^cGL^	94.40 ± 7.12^cGL^	11.48 ± 0.32^cGL^	83.78 ± 3.99^bGL^	nd	nd
BF	1130.73 ± 69.81^a^	94.34 ± 6.55^a^	4602.16 ± 297.45^a^	458.22 ± 24.61^a^	189.88 ± 8.06^a^	173.88 ± 3.06^a^	5.80 ± 0.25^a^	91.72 ± 3.72^a^	nd	nd
BFS	88536.80 ± 244.52^bDI^	1923.20 ± 19.37^bDI^	11343.60 ± 136.55^bDI^	6105.20 ± 81.06b^DI^	254.20 ± 0.86^bDI^	88.80 ± 2.88^bDI^	10.69 ± 0.17^bDI^	121.63 ± 2.16^bCI^	nd	nd
BFI	169.07 ± 7.38^cEL^	3276.00 ± 167.92^cFL^	19,491.00 ± 480.40^cHL^	10050.00 ± 345.36^cHL^	560.44 ± 35.52^cHL^	132.14 ± 12.93^cHL^	13.14 ± 0.43^cHL^	77.20 ± 1.62^cHL^	nd	nd
*Lamb*										
LP	1088.91 ± 14.42^a^	189.26 ± 6.53^a^	5063.43 ± 144.1^a^	500.25 ± 18.80^a^	92.66 ± 3.55^a^	664.86 ± 20.36^a^	8.40 ± 0.22^a^	78.06 ± 1.61^a^	nd	nd
LPS	81683.60 ± 583.92^bAJ^	1534.80 ± 6.61^bAJ^	8985.60 ± 52.56^bAJ^	2422.40 ± 16.05b^AJ^	234.40 ± 1.44^bAJ^	172.40 ± 1.86^bAJ^	11.12 ± 0.02^bAJ^	79.88 ± 0.14^bAI^	nd	nd
LPI	143.47 ± 17.03^cEM^	4125.00 ± 80.95^cEM^	24799.00 ± 490.51^cEM^	8413.70 ± 168.60^cEM^	110.11 ± 6.55^cEM^	515.01 ± 21.99^cEM^	15.20 ± 0.28^cEM^	73.44 ± 0.23^cEM^	nd	nd
LV	1310.90 ± 35.25^a^	198.59 ± 4.21^a^	5467.47 ± 89.78^a^	538.85 ± 19.36^a^	156.85 ± 5.07^a^	803.93 ± 20.01^a^	10.62 ± 0.42^a^	97.64 ± 2.73^a^	nd	nd
LVS	66403.60 ± 1635.17^bBJ^	1216.24 ± 9.50^bBJ^	7758.00 ± 76.96^bBJ^	2450.80 ± 37.99^bACI^	694.78 ± 8.75^bBJ^	144.01 ± 4.47^bBJ^	11.15 ± 0.08^bAJ^	69.60 ± 1.46^bBJ^	nd	nd
LVI	142.64 ± 10.66^cEX^	2949.90 ± 101.58^cFL^	17,771.00 ± 316.74^cFL^	9442.00 ± 377.82^cFL^	246.41 ± 17.96^cFM^	353.44 ± 18.15^cFM^	16.18 ± 0.09^cFM^	75.04 ± 1.3^1cFL^	nd	nd
LB	659.14 ± 18.05^a^	53.13 ± 1.46^a^	2450.94 ± 78.42^a^	265.20 ± 9.52^a^	181.56 ± 3.86^a^	849.03 ± 21.72^a^	8.58 ± 0.28^a^	101.22 ± 3.28^a^	nd	nd
LBS	68580.00 ± 544.88^bCJ^	611.20 ± 11.94^bCJ^	5622.00 ± 95.87^bCJ^	970.00 ± 41.52^bBJ^	970.00 ± 41.52^bCJ^	145.70 ± 10.07^bBJ^	10.23 ± 0.26^bBJ^	110.25 ± 1.05^bCJ^	nd	nd
LBI	113.16 ± 11.67^cFM^	2220.00 ± 177.12^cGM^	13,416.00 ± 181.96^cGM^	5668.00 ± 71.78^cGM^	728.40 ± 27.09^cGM^	310.04 ± 5.05^cGM^	16.23 ± 0.16^cFM^	115.14 ± 1.33^cGM^	nd	nd
LF	1106.86 ± 32.24^a^	92.62 ± 2.68^a^	4595.83 ± 119.97^a^	460.78 ± 15.30^a^	187.54 ± 2.99^a^	560.05 ± 16.81^a^	8.68 ± 0.23^a^	89.15 ± 2.52^a^	nd	nd
LFS	77037.60 ± 123.05^bDJ^	1545.20 ± 41.82^bAJ^	9236.00 ± 269.99^bDJ^	2479.80 ± 44.97^bCJ^	344.20 ± 13.69^bDJ^	159.11 ± 12.02^bCJ^	8.91 ± 0.12^aCJ^	64.12 ± 2.10^bDJ^	nd	nd
LFI	138.44 ± 14.11^cEM^	4234.00 ± 129.94^cEM^	25373.00 ± 764.10^cEM^	8839.00 ± 1221.99^cEFM^	486.46 ± 15.88^cHM^	343.34 ± 10.46^cHM^	13.91 ± 0.49^bGM^	60.55 ± 1.54^cHM^	nd	nd
*Chicken*										
CP	1745.47 ± 80.34^a^	293.68 ± 12.41^a^	5967.59 ± 417.23^a^	601.06 ± 35.36^a^	182.67 ± 7.83^a^	18.43 ± 0.69^a^	4.34 ± 0.17^a^	153.51 ± 9.08^a^	nd	nd
CPS	64401.20 ± 999.51^bAK^	1441.40 ± 53.98^bAI^	8612.00 ± 276.72^bAK^	2040.00 ± 115.22^bAK^	292.80 ± 19.84^bAK^	15.60 ± 3.50^bAK^	7.31 ± 0.09^bAK^	125.59 ± 7.85^bAJ^	nd	nd
CPI	118.24 ± 8.92^cEM^	3806.00 ± 95.25^cEL^	22892.00 ± 279.1^cEL^	8473.00 ± 221.90^cEM^	191.30 ± 1.99^cEN^	18.40 ± 2.15^cEN^	11.06 ± 0.03^cEM^	119.05 ± 0.51^bEN^	nd	nd
CV	1879.51 ± 129.43^a^	277.99 ± 4.97^a^	6201.33 ± 317.66^a^	637.14 ± 26.12^a^	281.60 ± 7.86^a^	16.67 ± 0.82^a^	8.58 ± 0.51^a^	136.28 ± 6.99^a^	nd	nd
CVS	82118.00 ± 402.50^bBK^	1284.40 ± 10.38^bBK^	7732.80 ± 39.66^bBJ^	2645.60 ± 23.70^bBJ^	673.40 ± 6.31^bBK^	12.40 ± 2.77b^BK^	9.17 ± 0.04^bBK^	100.76 ± 0.05^bBK^	nd	nd
CVI	142.46 ± 3.86^cFX^	3331.00 ± 28.08^cFM^	20,099.00 ± 154.70^cFM^	9426.00 ± 50.59^cFL^	292.64 ± 10.13^cFN^	16.60 ± 3.03^aEN^	12.85 ± 0.07^cFM^	108.70 ± 0.40^cFM^	nd	nd
CB	825.34 ± 39.63^a^	181.45 ± 6.4^a^	2455.13 ± 86.19^a^	265.71 ± 14.30^a^	204.22 ± 11.34^a^	22.52 ± 1.14^a^	7.14 ± 0.35^a^	168.39 ± 5.30^a^	nd	nd
CBS	74209.60 ± 151.01^bCI^	792.80 ± 9.33^bCK^	5712.40 ± 26.86^bCJ^	1249.60 ± 10.99^bCI^	756.80 ± 6.92^bCK^	14.40 ± 1.19^bABK^	7.96 ± 0.15^bCK^	120.52 ± 7.08^bACK^	nd	nd
CBI	150.84 ± 3.53^cGL^	2635.00 ± 322.39^cGL^	15,929.00 ± 407.35^cGL^	6807.00 ± 31.08^cGN^	504.66 ± 6.5^cGN^	25.10 ± 3.23^cFN^	13.55 ± 0.13^cGM^	112.93 ± 0.16^cGM^	nd	nd
CF	1578.90 ± 69.61^a^	295.06 ± 9.05^a^	5534.80 ± 180.43^a^	541.49 ± 35.07^a^	226.38 ± 11.24^a^	21.80 ± 1.13^a^	6.30 ± 0.29^a^	113.09 ± 2.30^a^	nd	nd
CFS	78656.80 ± 1812.78^bDK^	1564.40 ± 9.49^bDJ^	9344.40 ± 18.20^bDJ^	2291.60 ± 123.90^bDK^	406.80 ± 2.44^bDK^	12.80 ± 0.78^bABK^	7.53 ± 0.11^bDK^	114.84 ± 0.22^bCK^	nd	nd
CFI	121.74 ± 3.23^cEN^	4391.00 ± 3.77^cHN^	26417.00 ± 206.12^cHN^	8475.00 ± 120.64^cEM^	636.40 ± 12.93^cHN^	16.40 ± 1.65^cEN^	13.09 ± 0.13^cHL^	126.94 ± 0.47^cHN^	nd	nd
EFSA	2000 (mg)	350 (M) 300 (F) (mg)	3500 (mg)	550 (mg)	11–16 mg (RDA)	1.3–1.6 (RDA) mg	2–3 mg	7.5–12.7 (mg)		

*Note:* Mineral contents were determined in the soluble (supernatant) fraction obtained after centrifugation of the gastric and intestinal digests and represent the bioaccessible fraction. Lowercase letters a, b, c indicate differences in mineral contents among cooked, gastric‐, and intestinal‐digested samples within the same liver type; different letters denote significant differences at *p* < 0.05. Uppercase letters A, B, C and D indicate differences in mineral contents after gastric digestion among samples of the same liver type prepared by different cooking methods; different letters denote significant differences at p<0.05. Uppercase letters E, F, G and H indicate differences in mineral contents after intestinal digestion among samples of the same liver type prepared by different cooking methods, with different letters denoting significant differences *p* < 0.05. Uppercase letters I, J, and K indicate differences in mineral contents after gastric digestion among different liver types cooked by the same cooking method; different letters denote significant differences at *p* < 0.05. Uppercase letters L, M, and N indicate differences in mineral contents after intestinal digestion among different liver types cooked by the same method; different letters denote significant differences at *p* < 0.05. X indicates that no statistically significant differences was found among the compared samples (*p* > 0.05).

Abbreviations: BB, beef boiling; BBI, beef boiling intestine; BBS, beef boiling stomach; BF, beef deep frying; BFI, beef deep frying intestine; BFS, beef deep frying stomach; BP, beef pan‐frying; BPI, beef pan‐frying intestine; BPS, beef pan‐frying stomach; BV, beef sous‐vide; BVI, beef sous‐vide intestine; BVS, beef sous‐vide stomach; CB, chicken boiling; CBI, chicken boiling intestine; CBS, chicken boiling stomach; CF, chicken deep frying; CFI, chicken deep frying intestine; CFS, chicken deep frying stomach; CP, chicken pan‐frying; CPI, chicken pan‐frying intestine; CPS, chicken pan‐frying stomach; CV, chicken sous‐vide; CVI, chicken sous‐vide intestine; CVS, chicken sous‐vide stomach; EFSA, European Food Safety Authority; F, female; LB, lamb boiling; LBI, lamb boiling intestine; LBS, lamb boiling stomach; LF, lamb deep frying; LFI, lamb deep frying intestine; LFS, lamb deep frying stomach; LP, lamb pan‐frying; LPI, lamb pan‐frying intestine; LPS, lamb pan‐frying stomach; LV, lamb sous‐vide; LVI, lamb sous‐vide intestine; LVS, lamb sous‐vide stomach; M, male; nd, not detected; RDA, Recommended Dietary Allowance.

The bioaccessibility of minerals depends not only on their total content in foods but also on the fractions released into the soluble phase during digestion. Therefore, in vitro gastrointestinal digestion models are employed as a crucial tool for assessing the relative bioaccessibility potential of minerals under simulated digestion conditions. A marginal increase in the Na concentration of the samples was observed after gastric digestion. Following intestinal digestion, Na levels ranged from 113.16 ± 11.67 to 186.01 ± 34.14 mg/kg. No significant differences were found among liver types cooked by sous‐vide (*p* > 0.05), whereas deep frying resulted in significant inter‐type variations (*p* < 0.05). The lowest reduction relative to initial values (75.90%–82.83%) after intestinal digestion was observed in boiled liver samples across all liver types. The minerals Mg, K, and P exhibited marginal increases in all liver samples following both gastric and intestinal digestion. In the gastric phase, the highest Mg concentrations were detected in deep fried beef, lamb, and chicken samples, while in the intestinal phase, Mg was highest in deep fried lamb and chicken samples and in pan‐fried beef samples. Overall, the highest Mg content was observed in the BFI sample for the stomach (1923.2 ± 19.37 mg/kg) and in the CFI sample for the intestine (4391 ± 3.77 mg/kg). A similar distribution was observed for potassium; with the highest values detected in the gastric phase of deep fried samples. In contrast, the highest concentrations were found in the intestinal phase of deep fried lamb and chicken samples, as well as in pan‐fried beef samples. The highest K concentration was recorded in the BFS sample for the stomach (11343.6 ± 136.55 mg/kg), and in the CFI sample for the intestine (26,417 ± 206.12 mg/kg). For the phosphorus (P) mineral, the highest gastric value was observed in the BFS sample (6105.2 ± 81.06 mg/kg), whereas the highest intestinal value was found in the BPI sample (11,330 ± 433.73 mg/kg). The increases observed in the macrominerals sodium (Na), magnesium (Mg), potassium (K), and phosphorus (P) can be attributed to two primary mechanisms. Firstly, the simulated gastric and intestinal fluids and buffer solutions employed in the in vitro digestion protocol are used to maintain pH stability and enzymatic activity, and they contain the minerals in question, thereby, contributing method‐derived mineral input to the digestion environment. Secondly, proteolytic enzymes activated during digestion, along with pH variations, facilitate the breakdown of proteins and other complex biomolecules in liver tissue, thereby enabling the release of minerals that are bound within intracellular and matrix structures. The increase of these macrominerals, which are both incorporated into the cellular structure and bound to complex macromolecular components, following gastric and intestinal digestion can be attributed to the effect of in vitro digestion in enhancing the breakdown of the matrix and consequently releasing the bound mineral content.

Fe content increased in all samples after gastric digestion, with the highest rise and maximum concentration detected in the LBS (970 ± 41.52 mg/kg). Cooking methods induced significant differences in Fe levels both within liver types and across the same cooking method applied to different liver types (*p* < 0.05). Boiling and sous‐vide were particularly effective in enhancing Fe content during the gastric phase in all liver types. Following intestinal digestion, Fe levels were higher than the initial values except in deep fried samples, while remaining lower than the gastric values. In BFI, LFI, and CFI samples, Fe continued to increase beyond the gastric phase. The highest intestinal Fe concentrations among chicken liver were detected in pan‐fried (191.3 ± 1.99 mg/kg) and sous‐vide (292.64 ± 10.13 mg/kg) treatments. Post‐digestion, BFI (560.44 ± 35.52 mg/kg), CFI (636.4 ± 12.93 mg/kg), and LBI (728.4 ± 27.09 mg/kg) exhibited the greatest overall Fe enrichment. It is noteworthy that iron, an essential mineral for human health, did not exhibit any reduction in its content after in vitro digestion under any of the applied thermal processing methods. This phenomenon is likely attributable to the thermal denaturation of the myoglobin molecule and the consequent increase in insoluble heme iron, which may have altered the ratio between heme and non‐heme iron (Wereńska et al. 2023).

Lamb liver exhibited the highest Cu content, followed by beef and chicken liver. In all samples, Cu content decreased following gastric digestion. Following gastric digestion, the highest values were observed in the BFS sample of beef liver (88.8 ± 2.88 mg/kg). In lamb and chicken, the peak concentrations were detected in pan‐fried samples, at 172.4 ± 1.86 and 15.6 ± 3.5 mg/kg, respectively. Although post‐intestinal digestion Cu levels remained below the initial values, they exhibited an increase compared to the post‐gastric values. In all three liver types, the lowest reductions following intestinal digestion were observed in the pan‐fried samples. At the same time, the highest values were recorded in the BPI, LPI, and CBI samples, measuring 155.1 ± 9.74, 515.01 ± 21.99, and 25.1 ± 3.23 mg/kg, respectively. Mn content increased after gastric and intestinal digestion in all samples except BVS. Post‐digestion, beef liver showed the highest Mn level in the pan‐fried sample (13.137 ± 0.43 mg/kg), while LBI and CBI reached 16.23 ± 0.16 and 13.55 ± 0.13 mg/kg, respectively. Pan‐frying yielded the most pronounced Mn enhancement after digestion, particularly in beef and chicken liver. Post‐digestion, the highest Zn concentrations were detected in LBI (115.14 ± 1.33 mg/kg) and CFI (126.94 ± 0.47 mg/kg), and only these values exceeded the initial levels. Overall, chicken liver exhibited the highest Zn content among all liver types and retained the highest Zn levels after digestion across all cooking treatments.

EFSA has established Dietary Reference Values (DRVs) for essential minerals, which serve as the primary benchmark for evaluating nutritional adequacy. In the present study, mineral levels determined after in vitro digestion were compared with EFSA reference values (EFSA 2017). To provide a realistic nutritional perspective, these findings were interpreted considering a typical cooked liver serving size of approximately 50–100 g. Post‐intestinal digestion Na contents (11.31–18.60 mg/100 g) indicate that liver contributes only marginally to daily sodium intake relative to EFSA recommendations (2000 mg/day), and its nutritional relevance for Na should therefore be considered within the context of the overall diet. In contrast, Mg contents (222.0–439.1 mg/100 g) suggest that a standard serving of liver may substantially contribute to daily Mg requirements, with some samples approaching or exceeding EFSA reference values. Potassium levels (1341.6–2641.7 mg/100 g) correspond to approximately 38%–75% of the daily recommended intake per 100 g, indicating a meaningful contribution depending on portion size. Iron concentrations (11.01–72.84 mg/100 g) markedly exceeded EFSA daily reference values, confirming liver as a concentrated dietary source of Fe; however, nutritional interpretation should be approached cautiously, considering portion size and bioaccessibility. Manganese contents (1.10–1.62 mg/100 g) provided approximately half of the recommended daily intake, while Zn levels (6.05–12.69 mg/100 g) indicated that a typical serving could substantially contribute to daily Zn requirements. Overall, these results confirm that liver represents a nutrient‐dense food with respect to essential minerals, although health‐related evaluations should account for realistic consumption portions and mineral bioaccessibility rather than concentration values alone. Interspecies comparisons should be interpreted with caution, as observed differences partly reflect intrinsic compositional characteristics of different liver types. High concentrations of certain minerals, particularly iron, further emphasize the need to consider portion size and consumption frequency when translating these findings into dietary recommendations.

### 3.5 In Vitro Bioaccessibility Index (BI%) of Soluble Mineral Fractions in Cooked Liver Samples Following Simulated Digestion

The Bioaccessibility Index (BI%), defined as the proportion of minerals released into the soluble intestinal fraction during in vitro digestion, showed significant differences in the planned comparisons among cooking methods within each liver type and among liver types within the same cooking method (*p* < 0.05), as presented in Table 5. The bioaccessibility of Na reached its highest levels after boiling in all three liver types; these values were 24.01% ± 2.38%, 17.14% ± 1.61%, and 18.21% ± 0.88% for beef, lamb, and chicken liver, respectively. From the perspective of Na bioaccessibility, beef liver subjected to pan‐frying, boiling, and deep frying demonstrated a comparative advantage. Mg, K, and P stood out due to their high BI% values. It should be noted that enzymatic solutions, buffers, and other digestive fluids used in the in vitro digestion model inherently contain certain minerals, which may contribute to the mineral content measured in the soluble fraction after digestion. Moreover, the increases in mineral concentrations observed during the gastric and intestinal phases can also be attributed to the release of endogenous minerals as a result of tissue degradation and matrix disruption during digestion. Therefore, BI% values exceeding 100% reflect an enhanced release of minerals into the soluble intestinal fraction during digestion and do not represent physiological absorption or bioavailability in vivo. Accordingly, several studies have emphasized that in vitro digestion media may inherently contain minerals such as Mg, K, and P, and this issue should be carefully considered when interpreting bioaccessibility calculations (Igual et al. 2023; Souza et al. 2023).

**TABLE 5:** Bioaccessibility Index (BI%) of minerals in beef, lamb, and chicken livers subjected to different cooking methods after in vitro digestion.

Sample	Na	Mg	K	P	Fe	Cu	Mn	Zn	Cr	Co
BP	16.54 ± 2.92^aA^	3304.62 ± 310.33^aA^	466.11 ± 24.14^aA^	3058.16 ± 172.36^aA^	119.09 ± 8.98^aA^	85.26 ± 7.60^aA^	256.97 ± 14.15^aA^	104.04 ± 3.29^aA^	nd	nd
BV	9.31 ± 1.29^bA^	1512.07 ± 159.23^bA^	310.31 ± 13.79^bx^	1688.67 ± 139.46^bA^	103.76 ± 13.29^bA^	67.18 ± 9.49^bA^	163.92 ± 6.24^bA^	60.53 ± 4.50^bA^	nd	nd
BB	24.01 ± 2.38^cA^	4249.80 ± 322.66^cA^	699.06 ± 45.16^cA^	3312.03 ± 214.48^cA^	231.51 ± 11.30^cA^	51.48 ± 4.35^cA^	250.80 ± 16.83^aA^	85.59 ± 6.20^cA^	nd	nd
BF	15.00 ± 1.05^aA^	3490.46 ± 320.89^aA^	425.01 ± 26.50^dA^	2199.51 ± 143.68^dA^	295.28 ± 19.73^dA^	75.97 ± 7.57^abA^	226.91 ± 11.57^cA^	84.28 ± 3.28^cA^	nd	nd
LP	13.15 ± 1.59^aB^	2180.88 ± 50.79^aB^	490.04 ± 13.27^aA^	1684.36 ± 75.39^aB^	118.81 ± 7.13^aA^	77.58 ± 4.87^aA^	181.17 ± 5.11^aB^	94.12 ± 1.92^aB^	nd	nd
LV	10.83 ± 0.67^bB^	1485.89 ± 52.38^bA^	325.12 ± 7.95^bx^	1755.55 ± 111.62^abA^	157.00 ± 12.06^bB^	43.93 ± 2.27^bB^	152.56 ± 5.56^bB^	76.91 ± 2.31^bB^	nd	nd
LB	17.14 ± 1.61^cB^	4180.16 ± 316.90^cA^	547.71 ± 11.66^cB^	2140.19 ± 88.41^cB^	400.97 ± 14.33^cB^	36.54 ± 1.17^cB^	189.35 ± 5.75^cB^	113.86 ± 3.74^cB^	nd	nd
LF	12.46 ± 1.01^abB^	4573.66 ± 148.92^dB^	552.46 ± 21.76^cB^	1920.56 ± 260.61^bB^	259.14 ± 7.03^dB^	61.25 ± 1.05^dB^	160.49 ± 7.30^dB^	67.97 ± 2.33^dB^	nd	nd
CP	6.78 ± 0.60^aC^	1297.60 ± 49.03^aC^	385.24 ± 24.98^aB^	1414.74 ± 96.73^aC^	104.72 ± 2.33^aB^	97.52 ± 9.21^aB^	254.95 ± 9.74^aA^	77.79 ± 4.33^aC^	nd	nd
CV	7.58 ± 0.42^bC^	1198.57 ± 22.03^aB^	324.89 ± 16.14^bx^	1481.77 ± 61.05^abB^	103.69 ± 3.23^aC^	99.93 ± 17.96^aC^	150.13 ± 8.47^bB^	79.94 ± 3.89^aB^	nd	nd
CB	18.21 ± 0.88^cB^	1453.14 ± 168.90^bB^	649.34 ± 22.06^cC^	2568.71 ± 136.10^cC^	247.56 ± 14.78^bC^	111.3 ± 14.34^aC^	190.30 ± 9.27^cB^	67.13 ± 2.00^bC^	nd	nd
CF	7.68 ± 0.42^bC^	1489.44 ± 43.11^bC^	477.72 ± 14.33^dC^	1570.79 ± 94.61^bC^	281.58 ± 14.61^cA^	75.21 ± 6.14^bA^	208.36 ± 10.71^dC^	112.29 ± 2.25^cC^	nd	nd

*Note:* Values represent mineral concentrations measured in the soluble fraction obtained after centrifugation of the intestinal digests. Lowercase letters indicate statistically significant differences (*p* < 0.05) in mineral bioaccessibility percentages among cooking methods within the same liver type following in vitro digestion. Uppercase letters indicate statistically significant differences (*p* < 0.05) in mineral bioaccessibility percentages among liver types subjected to the same cooking method following in vitro digestion. X indicates that no statistically significant difference was found among the compared samples (*p* > 0.05).

Abbreviations: BB, beef boiling; BF, beef deep frying; BP, beef pan‐frying; BV, beef sous‐vide; CB, chicken boiling; CF, chicken deep frying; CP, chicken pan‐frying; CV, chicken sous‐vide; LB, lamb boiling; LF, lamb deep frying; LP, lamb pan‐frying; LV, lamb sous‐vide; nd, not detected.

Each cooking method may differentially influence the release of nutrients, such as minerals (Bhat et al. 2020). Mg and K exhibited higher BI% values in all liver types following boiling and deep frying, whereas P showed higher BI% values in boiled samples. Iron bioaccessibility varied markedly across liver species and cooking methods in the planned group comparisons. The highest Fe BI% among the samples was observed in the LB sample (400.97 ± 14.33), whereas in beef and chicken liver samples, this value was most pronounced in deep fried specimens. For Cu, chicken liver showed the highest BI% values across all cooking methods (75.21% ± 6.14% to 111.3% ± 14.34%), whereas for Mn, beef liver exhibited the highest BI% values across all cooking treatments (163.92% ± 6.24% to 256.97% ± 14.15%). Different cooking methods were found to be most favorable for Zn BI% depending on the liver type. Da Silva et al. (2017) highlighted that, when comparing the percentage bioaccessibility values of raw and heat‐treated samples, cooking was effective in enhancing the bioaccessibility of minerals in bovine liver. Furthermore, it has been reported that cooking beef liver samples using the sous‐vide method results in relatively high mineral bioaccessibility. The same authors reported that, following sous‐vide cooking of bovine liver samples, the bioaccessibility values were 42.6% for K, 43.9% for Mg, 39.5% for Fe, 26.9% for Cu, and 36.3% for Zn. In general, thermal processing can promote the release of minerals from the food matrix by inducing protein denaturation and structural disruption, thereby increasing their presence in the soluble fraction during in vitro digestion. In this context, cooking can enhance the BI% of minerals by disrupting cellular structures, breaking down mineral–macromolecule complexes, and facilitating the solubilization of bound mineral fractions. Overall, the BI% values reported in this study should be interpreted as relative indicators of mineral solubilization under standardized in vitro conditions rather than as absolute measures of mineral absorption or nutritional adequacy in vivo.

## 4 Conclusion

Overall, the findings indicate that mineral retention and mineral release into the soluble fraction during in vitro digestion vary according to the cooking treatment applied and the liver type evaluated. The observed differences among cooking treatments indicate that mineral availability from liver depends not only on its initial mineral composition but also on the extent to which minerals are retained during cooking and solubilized during digestion.

The sous‐vide method, due to its low temperatures and oxygen‐free conditions, minimized cooking losses and maintained higher mineral retention compared to other methods. Trace minerals, especially Fe, Cu, and Zn, were better preserved with sous‐vide, accompanied by higher BI% values reflecting enhanced mineral solubilization during in vitro digestion. Boiling caused the greatest losses of water‐soluble minerals (Na, K, Mg, P) due to leaching into the cooking water, while certain trace elements (Fe, Cu, Zn) showed increased concentrations following digestion. Deep frying and pan‐frying led to moderate reductions in macromineral content, with Mg showing the most pronounced decrease.

The in vitro digestion results highlighted the importance of bioaccessible (soluble) mineral fractions rather than total mineral content alone. Fe content did not decrease after digestion under any of the applied cooking methods; on the contrary, it generally exhibited an increasing trend, indicating effective release of iron into the soluble fraction during digestion, thereby confirming liver as a valuable dietary source of this mineral. Liver type was also associated with differences in BI% values; Na, Mg, P, and Mn BI% values were notably higher in beef liver, whereas K BI% was predominant in lamb liver. Fe BI%, although influenced by the cooking method, was pronounced in both beef and lamb livers, while Cu BI% was particularly high in chicken liver. These findings suggest that mineral release behavior during digestion differs according to both cooking treatment and liver type.

In conclusion, sous‐vide emerged as the most effective cooking method for liver in terms of preserving mineral content and enhancing mineral solubilization under in vitro digestive conditions. The evaluation of mineral retention using true retention coefficients allowed the differentiation between apparent concentration changes caused by moisture loss and actual mineral preservation following thermal processing. The high mineral content and elevated BI% values of liver highlight its importance as a nutrient‐dense food source. However, these findings should be interpreted as indicators of relative mineral release rather than direct measures of physiological absorption. Within this context, the results provide a scientific basis suggesting that cooking methods may affect the mineral nutritional quality of liver and that liver type should be considered when evaluating its potential contribution to dietary mineral intake. To validate the nutritional relevance of the in vitro results, further in vivo studies are recommended, considering different liver types, cooking methods, and realistic consumption scenarios.

## Author Contributions

**Osman Sagdic:** validation, conceptualization. **Hatice Bekiroglu:** formal analysis, validation, resources, data curation, visualization, investigation. **Muhammet Arici:** validation, conceptualization. **Ayşe Semra Aksoy:** writing – original draft, writing – review and editing, methodology, supervision, software, formal analysis, data curation, resources, conceptualization, visualization, validation, investigation.

## Ethics Statement

The authors have nothing to report.

## Conflicts of Interest

The authors declare no conflicts of interest.

## Data Availability

The data that support the findings of this study are available from the corresponding author upon reasonable request.
